# *Akkermansia muciniphila* participates in the host protection against helminth-induced cardiac fibrosis via TLR2

**DOI:** 10.1371/journal.ppat.1011683

**Published:** 2023-10-03

**Authors:** Jiaqi Wang, Xiaolei Liu, Ruohang Sun, Hanhai Mao, Mingyuan Liu, Xuemin Jin

**Affiliations:** 1 State Key Laboratory for Zoonotic Diseases, Key Laboratory for Zoonosis Research of the Ministry of Education, Institute of Zoonosis, College of Animal Sciences, Jilin University, Changchun, China; 2 State Key Laboratory for Zoonotic Diseases, Key Laboratory for Zoonosis Research of the Ministry of Education, College of Veterinary Medicine, Jilin University, Changchun, China; 3 Jiangsu Co-innovation Center for Prevention and Control of Important Animal Infectious Diseases and Zoonoses, Yangzhou, China; University of Medicine & Dentistry New Jersey, UNITED STATES

## Abstract

Helminth *Trichinella spiralis* (Ts) is one of the major pathogens of human infective myocarditis that can lead to cardiac fibrosis (CF). The gut microbiota involved in this pathology are of interest. Here, we use mice infected with Ts as a model to examine the interactions between gut microbes and host protection to CF. Infected mice show enhanced CF severity. We find that antibiotics treatment to deplete the microbiota aggravates the disease phenotype. Attempts to restore microbiota using fecal microbiota transplantation ameliorates helminth-induced CF. 16S rRNA gene sequencing and metagenomics sequencing reveal a higher abundance of *Akkermansia muciniphila* in gut microbiomes of Ts-infected mice. Oral supplementation with alive or pasteurized *A*. *muciniphila* improves CF via TLR2. This work represents a substantial advance toward our understanding of causative rather than correlative relationships between the gut microbiota and CF.

## Author summary

Cardiac fibrosis (CF) is related to almost all types of cardiovascular diseases. Parasitic helminths are important cause of mortality and morbidity in fibrosis. Emerging evidence has supported the protective effects of the gut microbiota in regulating host susceptibility to cardiovascular diseases. However, whether and how modulation of the gut microbiota contributes to protection against helminth-induced CF remain unknown. We established a mouse model of helminth-induced CF with *Trichinella spiralis* (Ts) infection. Loss of gut microbiota by antibiotics treatment aggravated helminth-induced CF. Restoration of gut microbiome alleviated helminth-induced CF, indicating gut microbiota could serve as a therapeutic target for helminth-induced CF. Increased abundances of *Akkermansia* genus and *Akkermansia muciniphila* species were found in the Ts-infected samples. Treatment of pasteurized A. muciniphila could positivley influence the outcomes of helminth-induced CF via TLR2. Our research indicates the important role of gut microbiota in protecting against helminth-induced CF and serves as a basis for other infectious CF.

## Introduction

The gut microbiota that inhabits the gastrointestinal tract affects health by regulating resistance to a range of diseases [1]. Several studies have led to the development of the "gut hypothesis in heart failure," which states that dysbiosis of the gut microbiota may contribute to adverse outcomes in heart disease patients [2–5]. Cardiac fibrosis (CF) is related to almost all types of cardiovascular diseases. Excessive CF leads to adverse cardiac remodeling and ultimately leads to heart failure [6]. CF has become an important global health problem for which there are limited treatment options [7]. Probiotics targeting the gut microbiota have been demonstrated to reduce myocardial infarct size, atherosclerotic plaque area and the incidence rates of postinfarction myocardial hypertrophy and heart failure [8–10]. Given these observations, understanding the role of gut bacterial contributions to the development of CF is of interest.

Infections with pathogens (*e*.*g*., viruses and helminths) are important cause of mortality and morbidity in fibrosis [11–15]. Host immune response controls helminth infection but can lead to fibrosis when improperly controlled [16]. Helminth *Trichinella spiralis* (Ts) is also one of the major pathogens of human infective myocarditis that can lead to CF [17]. In the hosts larvae penetrate the small intestine. Following maturation and mating, female worm residing in the intestine produce many larvae which then migrate into the bloodstream finally encysting in striated muscle. Each cyst then becomes fully encapsulated within striated skeletal muscle, but not occur in cardiac tissue [18]. A case report on nine fatal trichinosis cases shows that all cases are died in CF-related myocarditis. This report finds Ts only in skeletal muscle, but not in heart by histopathology [19]. Thus, there may be some other indirectly effect yet to be discovered about Ts-induced cardiac disease. Parasitic helminths of humans and animals and the resident gut microbial flora is attracting increasing attention due to their impact on the pathophysiology of parasitic infections and diseases [20–22]. It is now clear that microbiota compositional changes occur during infection with a variety of helminth species [21,23]. However, the potential impact of the helminth-altered gut microbiome on progression of helminth-induced CF is unknown. The causal relationship between these two features also remains unexplored.

In this paper, CF was observed in a mouse model of Ts infection. We explored the role of gut microbiome during helminth infection in the development of CF. Fecal microbiota transplant (FMT) of helminth-altered gut microbiota cannot drive CF. However, we found that FMT from healthy mice to infective mice provide a benefit to CF during Ts infection, indicating that manipulating the gut microbiome could be therapeutic strategy for treating helminth-induced CF. Using metagenomic analyses, we revealed a significant bacterial component of Ts-induced gut microbiota. Last, we also determined the intervention effect of a dominant probiotic on the severity of CF.

## Results

### Predepletion of gut microbiota aggravated helminth-induced CF

**Fig 1:**
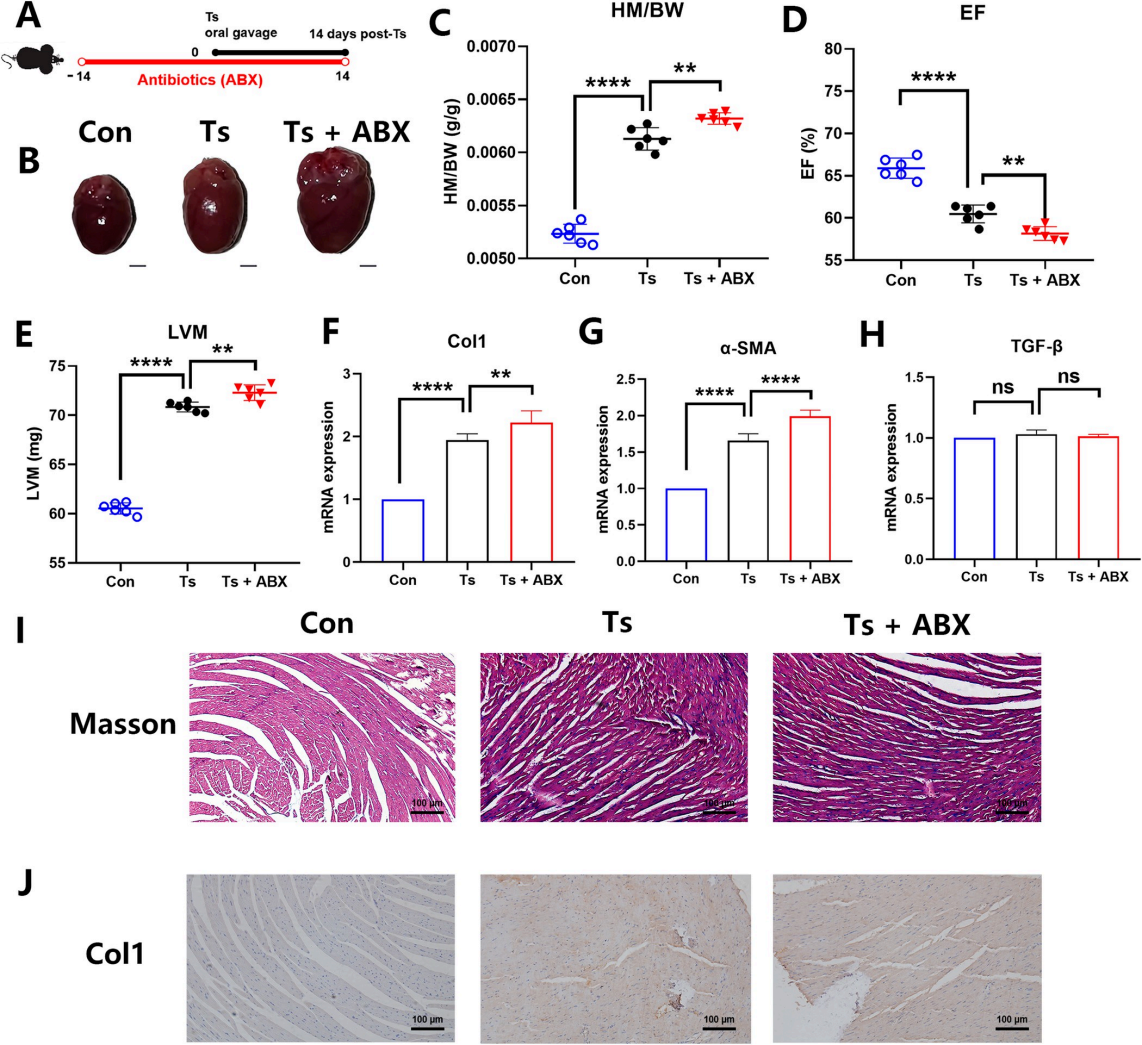
Loss of the gut microbiota aggravated helminth-induced cardiac fibrosis. (A) Experimental scheme of Ts-infected mice received antibiotics (ABX) treatment. (B) Whole heart images of the controls (Con) (n = 6), Ts-infected mice (Ts) (n = 6) and Ts-infected mice with antibiotics (Ts+ABX) (n = 6). Representative images are shown. (C) Heart mass-to-body weight ratio (HM/BW). (D and E) Ejection fraction (EF) and left ventricular weight (LVM) obtained by cardiac ultrasound. (F, G and H) qPCR analysis of Collagen-1 (Col1), α-SMA and TGF-β expression in the heart tissue of mice. (I and J) Masson staining and Col1 immunohistochemistry results of heart tissues. Magnification, 200×. Scale bars, 100 μm. Representative images are shown. Data are shown as individual data points and mean ± SD. Data were compared by one-way ANOVA followed by Tukey multiple comparison tests. ns, not significant; *, p < 0.05; **, p < 0.01, ***, p <0.001, ****, p <0.0001.

We established a mouse model of helminth-induced CF with Ts infection (Fig 1A). Helminth infection induced left ventricular hypertrophy (Fig 1B), increasing in the heart mass-to-body weight ratio (HM/BW) (Fig 1C), decreasing in ejection fraction (EF) (Fig 1D) and increasing left ventricular mass (LVM) (Fig 1E). Collagen-1 (Col1) and α-SMA (markers of CF) mRNA expression were significant elevated in heart of mice infected with Ts (Fig 1F and 1D). However, there was no significant difference in the mRNA expression of TGF-β among all groups (Fig 1H). Compared with the control group, we observed an increase in interstitial collagen deposition by Masson trichrome staining during helminth infection (Fig 1I). This result was also confirmed by the immunohistochemical staining for Col1 (Fig 1J).

**Fig 2:**
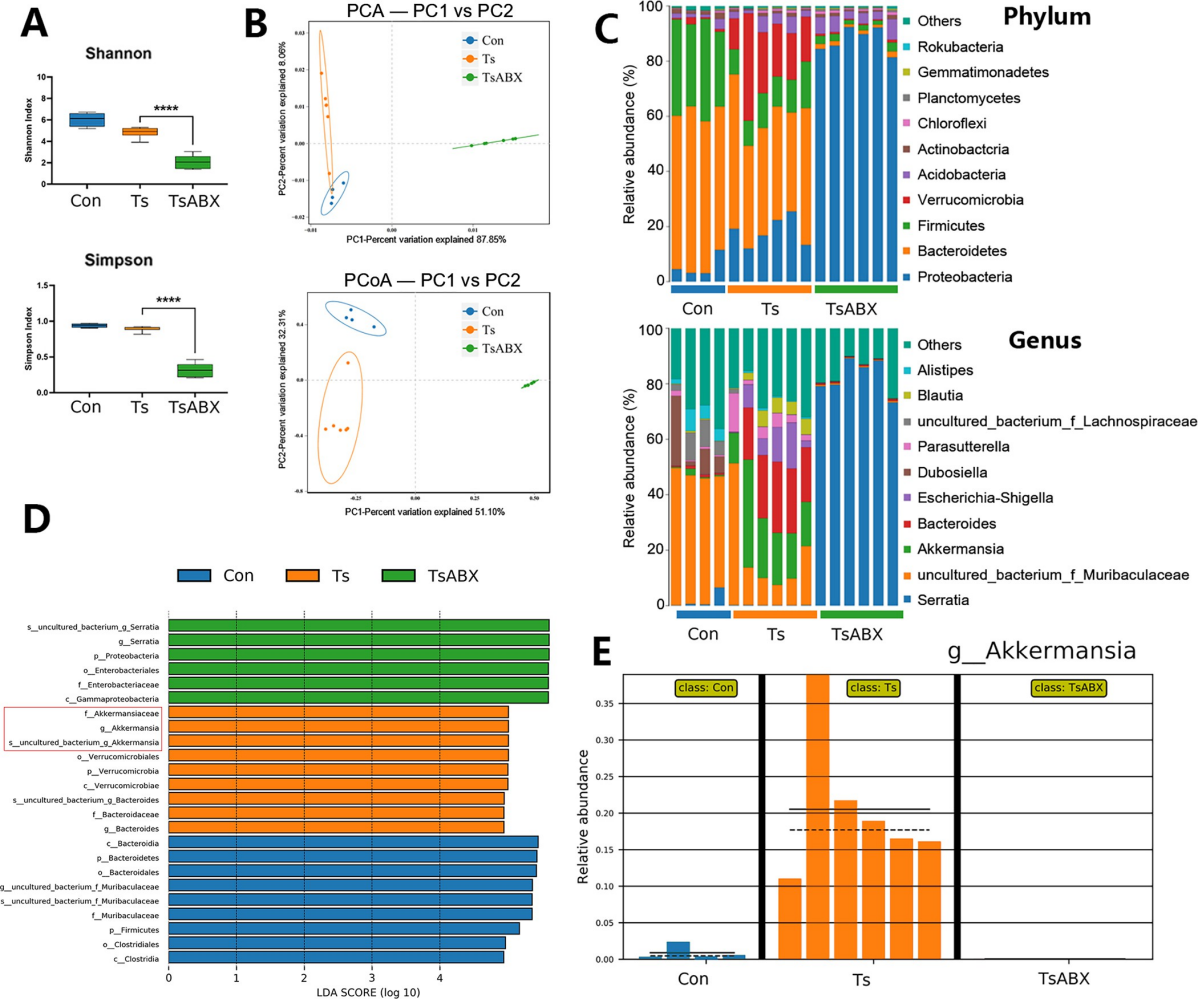
Characterization of the gut microbiota dysbiosis in Ts-infected mice with antibiotics (ABX) treatment using 16s rRNA sequencing. (A) α diversity (Shannon index and Simpson index) in the microbiota from the controls (Con), Ts-infected mice and Ts-infected mice with antibiotics treatment (TsABX). (B) Principal component analysis (PCA) and principal coordinates analysis (PCoA) plot (β diversity). (C) Phylum and genus-level median relative abundances. (D) Linear discriminant analysis (LDA) effect size showing the most significantly differentially abundant taxa enriched in the microbiota. (E) Comparison proportion of genus levels of *Akkermansia* based on 16S rRNA gene sequencing.

However, Ts larvae were not detected by artificial digestion of heart and not observed in the heart tissues by histopathology daily from 1 to 14 dpi, indicating Ts-induced CF is not directly caused by heart parasitism. Given that dysbiosis of the gut microbiota may contribute to adverse outcomes in heart disease [2–5], we explored the role of gut microbiota in helminth-induced CF. We depleted the gut microbiota of Ts-infected mice using a broad antibiotics (ABX) regiment, as previously decribed [24]. α-diversity can be affected by species richness (i.e., the number of unique species/OTUs) and the abundance of each species. The ABX treatment significantly reduced gut microbial diversity and richness shown by α-diversity analyses (Shannon index and Simpson index) (Fig 2A), indicating that the ABX treatment could effectively abolish gut microbiota. β-diversity was significantly different among all groups (Fig 2B). This indicates that the gut microbiome undergoes dramatic fluctuations compared to control. Moreover, although there was no significant difference of muscle larvae burden after the ABX treatment (S1 Fig), we found that the above symptoms were significantly aggravated (Fig 1) after ABX treatment. These findings indicate that these commensal microbes may be involved in the development of helminth-induced CF.

To further characterize differences, we performed 16S rRNA analyses of the gut microbiome. We carried out a comparison in the controls, Ts group and Ts+ABX group of the relative abundance of bacterial taxa. We found that greater abundance of phylum *Verrucomicrobia* was associated with Ts infection, as well as its genus *Akkermansia* (Fig 2C). Over-represented and under-represented features were identified using linear discriminant analysis effect size (LEfSe), which is based on Line Discriminant Analysis (LDA) scores also highlighted that Ts-infected mice had higher abundances of *Akkermansia* compared with the other groups (Fig 2D). The relative abundance of *Akkermansia* in all samples from three groups displayed an obvious higher level of this bacteria in the Ts group (Fig 2E).

### Restoration of gut microbiome alleviated helminth-induced CF

To further verify the causal relationship between the gut microbiota and CF during helminth infection, we transplanted feces from Ts-infected mice into the ABX-treated mice. We confirmed that there was no worm burden in the intestine of Ts-infected mice at 14 days post infection (dpi), as we have documented in previous study [25]. With this experiment, the influence of helminth can be ruled out through transplanting the feces from mice at this stage of infection. After FMT of Ts-altered gut microbiota, we found that there were no significant differences in left ventricular hypertrophy, the HM/BW, the EF or LVM between the control mice and the mice receiving FMT of Ts-altered microbes (FMT-Ts) (S2A–S2E Fig). The mRNA expression levels of Col1 and α-SMA, Masson’s trichrome staining and Col1 immunohistochemistry results were also not significantly different (S2E–S2I Fig), suggesting that Ts-altered gut microbiota alone cannot cause CF.

**Fig 3:**
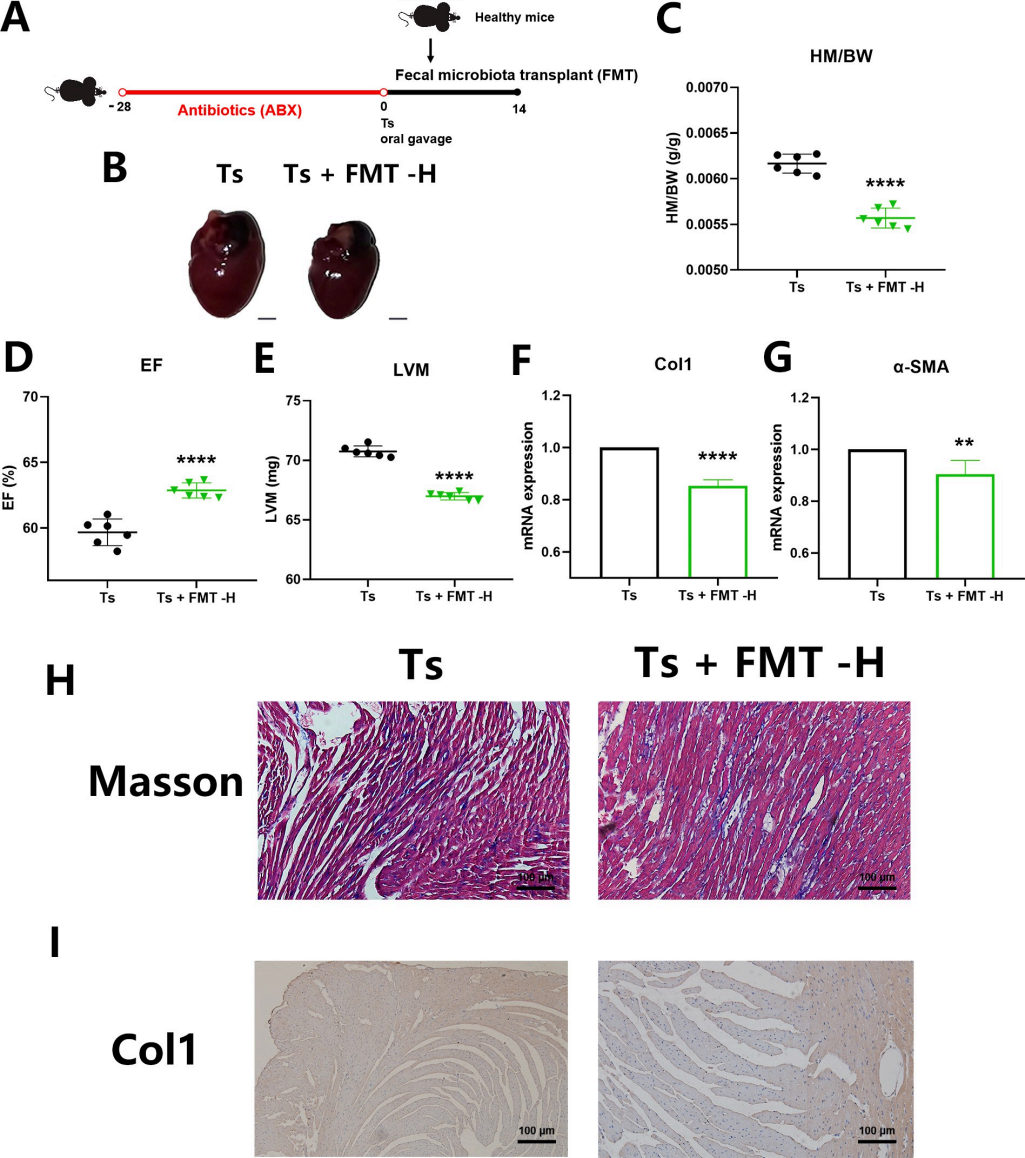
Restoration of gut microbiome alleviated helminth-induced cardiac fibrosis. (A) Experimental scheme of Ts-infected mice received healthy gut microbiota. (B) Whole heart images of Ts-infected mice (Ts) (n = 6) and Ts-infected mice received healthy fecal microbiota transplantation (FMT) (Ts+FMT-H) (n = 6). Representative images are shown. (C) Heart mass-to-body weight ratio (HM/BW). (D and E) Ejection fraction (EF) and left ventricular weight (LVM) obtained by cardiac ultrasound. (F and G) qPCR analysis of Collagen-1 (Col1) and α-SMA expression in the heart tissue of mice. (H and I) Masson staining and Col1 immunohistochemistry results of heart tissues. Magnification, 200×. Scale bars, 100 μm. Representative images are shown. Data are shown as individual data points and mean ± SD. Statistical significance is calculated using paired student t-test. ns, not significant; *, p < 0.05; **, p < 0.01, ***, p <0.001, ****, p <0.0001.

Next, we investigated whether the gut microbiota can serve as regulatory targets to alleviate the progression of helminth-induced CF. We transplanted feces from the healthy mice into mice with helminth infection after gut microbiota clearance (Fig 3A). We showed that the increase in left ventricular hypertrophy and the HM/BW induced by Ts was significantly attenuated by FMT (Fig 3B and 3C). When compared to those of the Ts group, FMT-H increased the EF and reduced the LVM (Fig 3D and 3E) and it reduced mRNA expression of Col1 and α-SMA (Fig 3F and 3G). Masson’s trichrome staining and Col1 immunohistochemistry results indicated that FMT-H prevented the excessive collagen deposition (Fig 3H and 3I). We hypothesized that the gut microbiota could serve as a therapeutic target for helminth-induced CF.

### The changes in the gut microbiome signature

**Fig 4:**
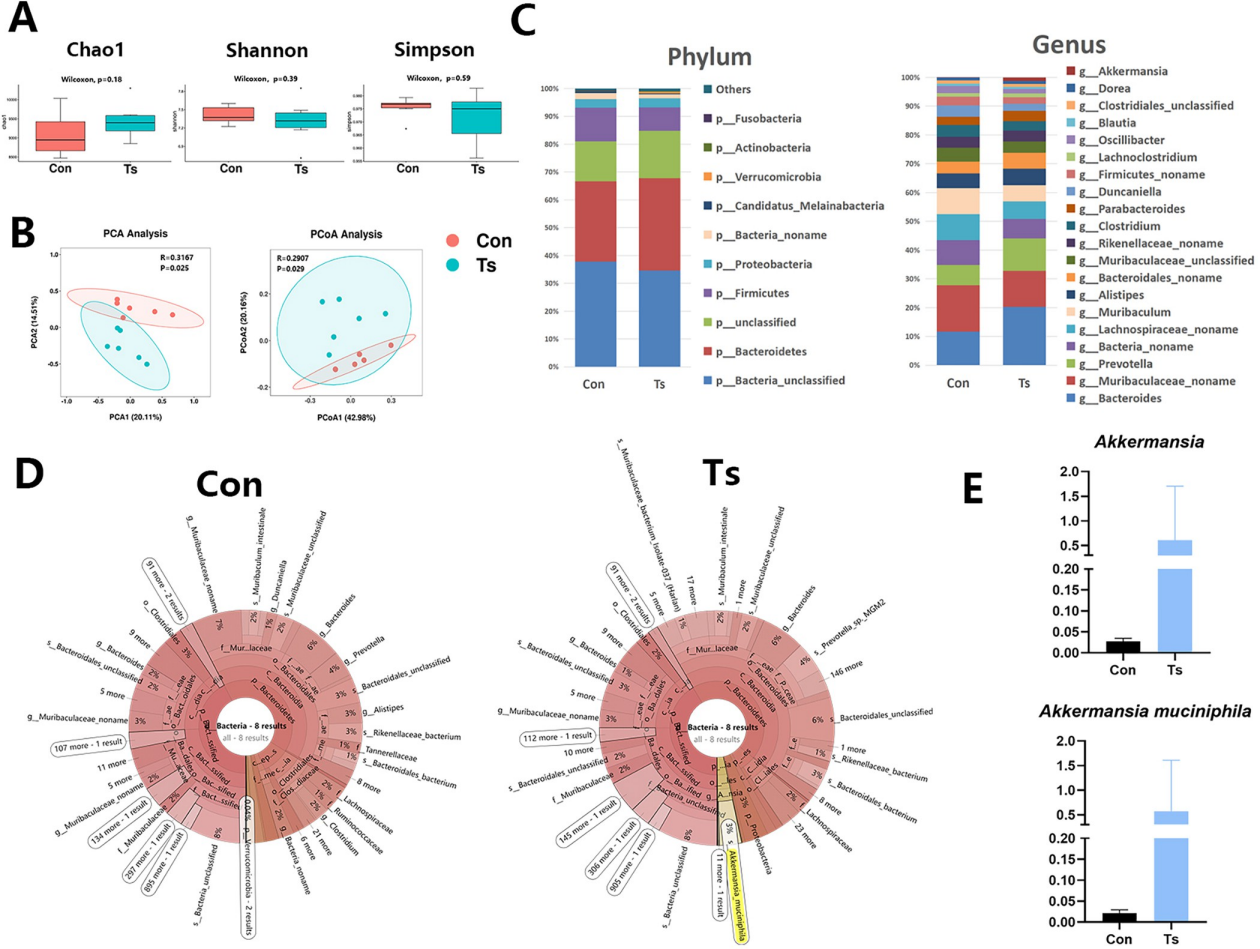
Characterization of the gut microbiota in Ts-infected mice using metagenomic sequencing. (A) Chao1 index, Shannon index and Simpson index was analyzed by metagenomic. Data are shown as individual data points and mean ± SD. Difference was tested with Wilcoxon signed-rank test. (B) Principal component analysis (PCA) and principal coordinates analysis (PCoA) plot (β diversity) in the microbiota from the controls (Con) (n = 6) and Ts-infected mice (n = 6). (C) Phylum-level and genus-level median relative abundances of Con and Ts group. (D) Krona plot of identified bacteria species of Con and Ts group. (E) Comparison proportion of genus and species levels of *Akkermansia muciniphila* based on metagenomics.

Unlike traditional 16S rRNA amplicon sequencing, metagenomics enables species-level analysis of community composition. Thus, we further identified bacterial species in helminth-altered gut microbiome using metagenomics. First, to assess the differences of bacterial diversity, sequences were aligned for alpha-diversity. The results showed that fecal microbial alpha-diversity (Chao1 index, Shannon index and Simpson index) were not significantly different between the control group and the Ts group (Fig 4A). To analyze microbiome space between samples, beta diversity was calculated using principal component analysis (PCA) and principal coordinates analysis (PCoA). The results presented a significantly different distribution between two groups (Fig 4B), suggesting that the control group has unique diversity and microbial distance metric from the Ts group. In general, there were four significantly different phyla, of which *Firmicutes* was high in the control group, *Bacteroidetes* and *Verrucomicrobia* were enriched in the Ts group (Fig 4C). At the genus level, *Muribaculaceae* and *Muribaculum* were significantly enriched in the control group compared to the Ts group. Moreover, *Bacteroides*, *Prevotella* and *Akkermansia* were significantly enriched in the Ts group compared to control (Fig 4C). The results of Krona plots demonstrated that *Akkermansia muciniphila* was one of the most dominant species (3% of bacteria) in the Ts-associated microbiome in the Ts-infected samples, while this species was almost undetected in the controls (Fig 4D). Increased abundances of *Akkermansia* genus and *A*. *muciniphila* species were also found in the Ts-infected samples ((Fig 4E)).

To understand the functional characteristics of the enriched metabolites, all the clean reads from metagenomic sequencing were aligned to the suggested database to obtain Kyoto Encyclopedia of Genes and Genomes (KEGG) modules enrichment from bacterial species. The function and KEGG metabolic pathway of the gut microbiota in the Ts group were obviously different from those in the control group. We found that the KEGG modules involved in citrate cycle (TCA cycle) and oxidative phosphorylation were overrepresented in the Ts group compared to control (S3 Fig).

### Both alive and pasteurized *A*. *muciniphila* had beneficial effect on helminth-induced CF

**Fig 5:**
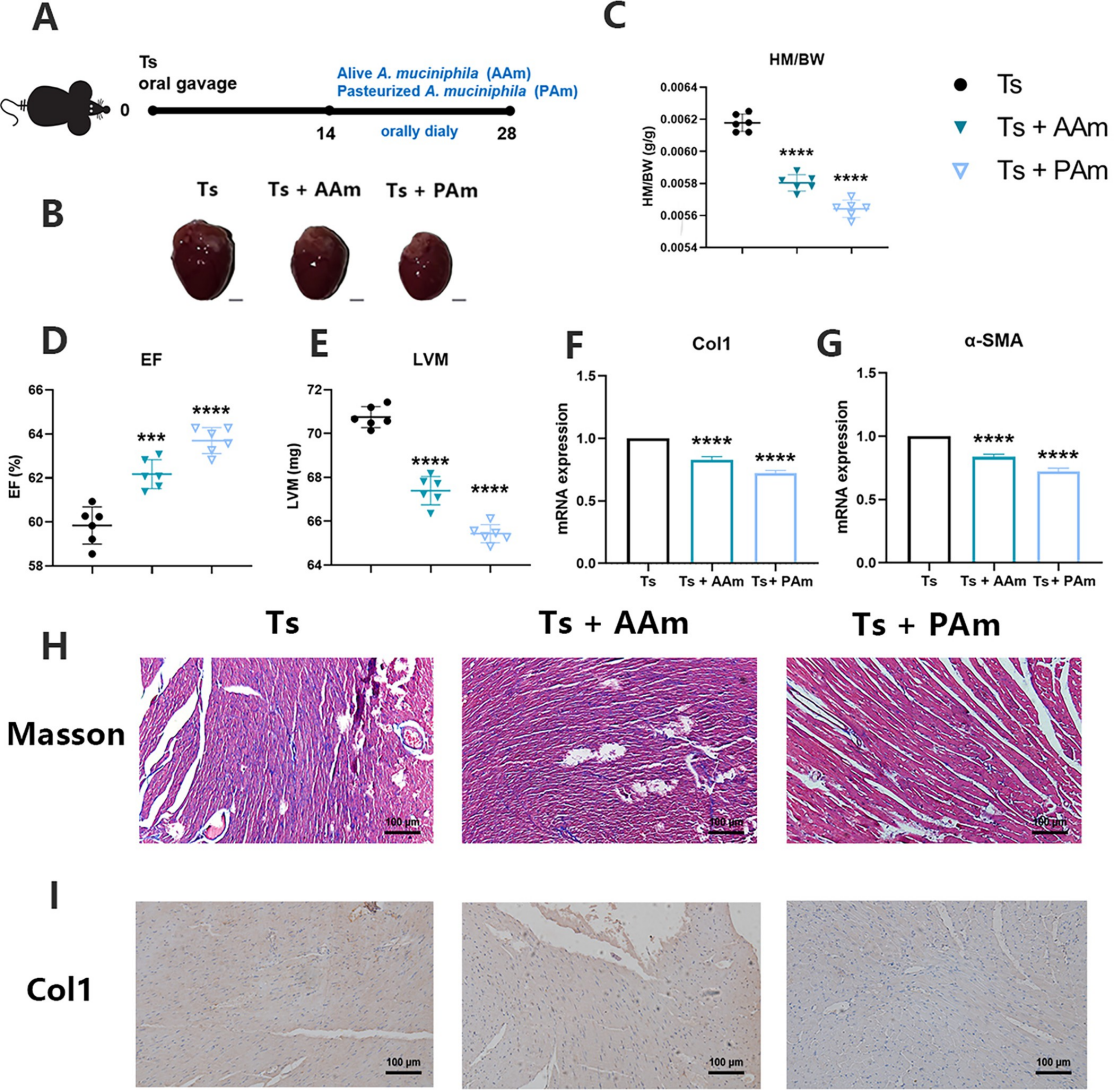
Both alive and pasteurized *Akkermansia muciniphila* had beneficial effect on helminth-induced cardiac fibrosis. (A) Experimental scheme of alive or pasteurized *A*. *muciniphila* administration (8 × 10^8^ CFU per dose orally) after Ts infection. (B) Whole heart images of Ts-infected mice (Ts) (n = 6) and Ts-infected mice treated with alive *A*. *muciniphila* (Ts+AAm) (n = 6) or pasteurized *A*. *muciniphila* (Ts+PAm) (n = 6). Representative images are shown. (C) Heart mass-to-body weight ratio (HM/BW). (D and E) Ejection fraction (EF) and left ventricular weight (LVM) obtained by cardiac ultrasound. (F and G) qPCR analysis of Collagen-1 (Col1) and α-SMA expression in the heart tissue of mice. (H and I) Masson staining and Col1 immunohistochemistry results of heart tissues. Magnification, 200×. Scale bars, 100 μm. Representative images are shown. Data are shown as individual data points and mean ± SD. Data were compared by one-way ANOVA followed by Tukey multiple comparison tests. ns, not significant; *, p < 0.05; **, p < 0.01, ***, p <0.001, ****, p <0.0001.

We considered that the dominant species *A*. *muciniphila* in the gut microbiota of mice with Ts infection played either a detrimental or beneficial role in the development of Ts-induced CF. Emerging evidence highlights that *A*. *muciniphila* inactivated by pasteurization for 30 min at 70°C and their effects can be partially or completely retained [26,27]. In our study, we evaluated the efficacy of alive *A*. *muciniphila* (AAm) or pasteurized *A*. *muciniphila* (PAm) on helminth-induced CF (Fig 5A). We demonstrated that both alive and pasteurized probiotics can alleviate the increase in left ventricular hypertrophy and the HM/BW induced by helminth infection (Fig 5B and 5C). Treatment of probiotics increased the EF and reduced the LVM (Fig 5D and 5E). Compared to the Ts group, Col1 and α-SMA mRNA expression were significantly downregulated in probiotics-treated mice. Moreover, we also observed that the effect of PAm was better than that of AAm, as determined through inspection of markers (Fig 5B–5I). However, treatment of PAm after the period of intestinal infection could not reduce the burden of muscle larvae (S4 Fig), indicating this probiotic could affect other immune pathways instead of anti-helminth immunity.

### TLR2 participated in the protection of PAm in helminth-induced CF

**Fig 6:**
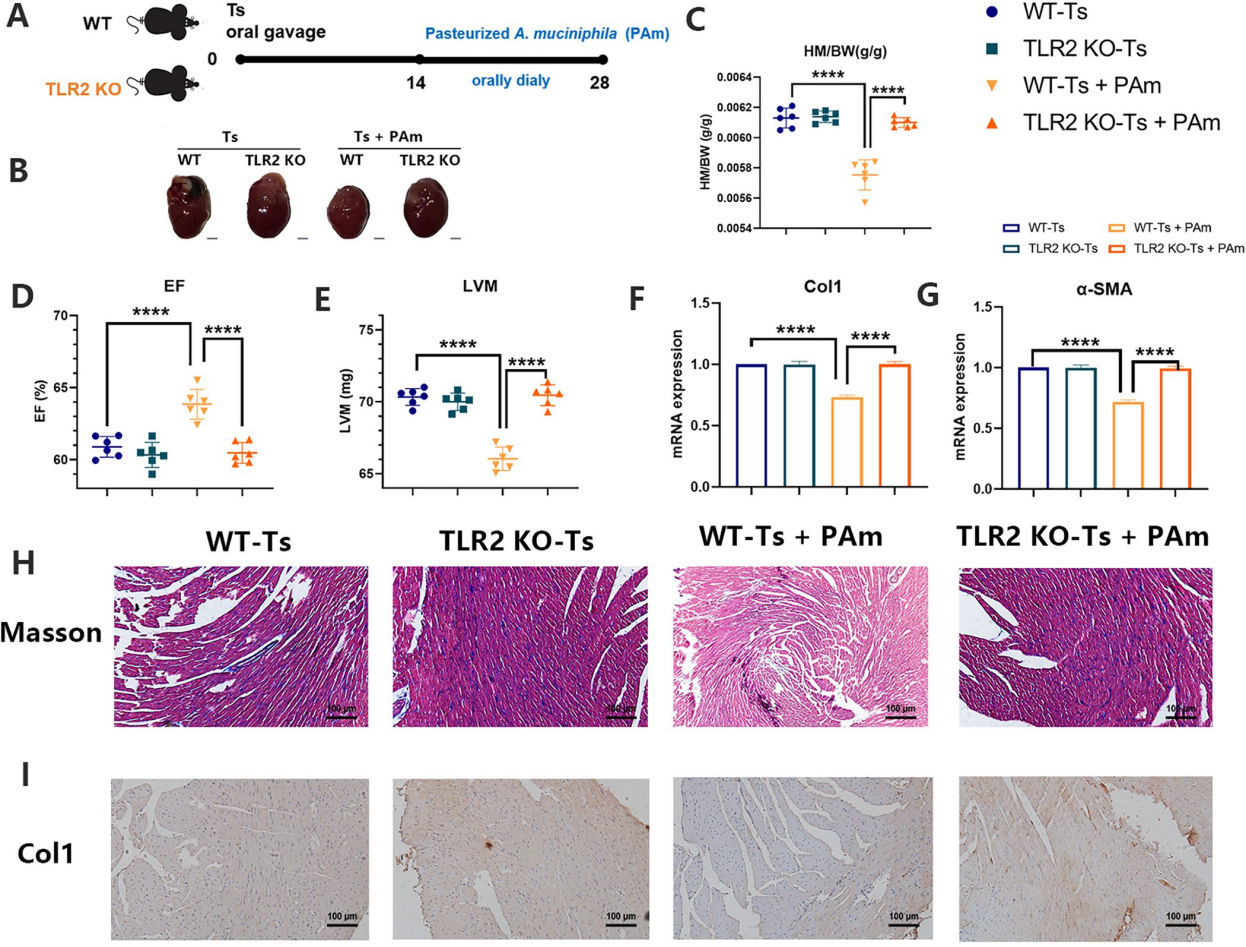
TLR2 participated in the protection of PAm against helminth-induced cardiac fibrosis. (A) Experimental scheme of pasteurized *A*. *muciniphila* administration (8 × 10^8^ CFU per dose orally) after Ts infection in wild type (WT) or TLR2 knockout (TLR2 KO) mice. (B) Whole heart images of Ts-infected WT or TLR2 KO mice (Ts) (n = 6) and Ts-infected mice treated with pasteurized *A*. *muciniphila* (Ts+PAm) (n = 6). Representative images are shown. (C) Heart mass-to-body weight ratio (HM/BW). (D and E) Ejection fraction (EF) and left ventricular weight (LVM) obtained by cardiac ultrasound. (F and G) qPCR analysis of Collagen-1 (Col1) and α-SMA expression in the heart tissue of mice. (H and I) Masson staining and Col1 immunohistochemistry results of heart tissues. Magnification, 200×. Scale bars, 100 μm. Representative images are shown. Data are shown as individual data points and mean ± SD. Data were compared by one-way ANOVA followed by Tukey multiple comparison tests. ns, not significant; *, p < 0.05; **, p < 0.01, ***, p <0.001, ****, p <0.0001.

A recent paper reports that *A*. *muciniphila* mediates the immunomodulatory function via regulation of the TLR2 [28]. In our study, we demonstrated that TLR2 deficiency did not interfere the severity of helminth-induced CF. There is no significant difference in all the markers for CF between the wild-type (WT) and TLR2 knockout (TLR2 KO) mice infected with Ts (Fig 6A–6H). However, treatment of PAm was unable to alleviate the pathology and markers of helminth-induced CF in TLR2 KO mice (Fig 6A–6H). These findings confirmed that TLR2 participated in the protection of PAm in CF during helminth infection, as expected.

## Discussion

The gut microbiota plays a fundamental role in the induction, education, and function of the host immune system, enabling the host to maintain its symbiotic relationship with microbiota. There is increasing attention on the complex interactions occurring between gastrointestinal parasitic helminths and the gut microbiota inhabiting the host gut [20, 29–32]. Enteric helminth infection induces potent immunomodulatory effects by direct regulation of host immunity or indirectly through alteration in gut microbiota. Helminth-induced immunomodulatory effects contribute to the development of pathological fibrosis in many different organ systems [17,33,34]. In our study, the gut microbiome altered by helminth *Trcihinella* cannot result in the pathological fibrosis in heart. This finding was unexpected. A potential explanation is the immunomodulation by *Trichinella* occurs independently of changes in the microbiota [29]. These results indicated that these alteration of microbial composition by *Trichinella* was not the main factor triggering helminth-induced CF.

Although gut microbiota was not confirmed as a direct pathogenic factors, dysbiosis and subsequent dysregulation of microbiota-related immunological processes affect the development of the disease [35–37]. Microbiota depletion in mice has been shown to be harmful during myocardial infarction [24]. Other models highlight the pathogenic role of gut microbiota-derived metabolite in CF [38]. Excessive kynurenine aggravates the severity of CF [39]. Trimethylamine-N-oxide (TMAO) supplementation induces cardiac hypertrophy and myocardial fibrosis [40]. Indeed, we observed that ABX-treated mice exhibited more severe CF during *Trichinella* infection, indicating that there are some pathogenic factors such as pathogenic microbiota and their metabolites in the development of helminth-induced CF. We further found that FMT involved transferring microbiota from healthy mice to Ts-infected mice could alleviate the severity of helminth-induced CF. The gut microbiome is comprised of mutualistic including pathogenic and beneficial microorganisms. Restoration of gut microbiota can result in a decrease of pathogenic bacteria and an increase of probiotics. In another model by transverse aortic constriction (TAC), gut microbiota reconstitution in the onset of TAC can also partially reverse cardiac pressure overload [41]. Manipulation of the microbiome is generally considered to have great potential for developing new therapies and finding preventive measures of complex diseases in humans. We proved the correlation between gut microbiota and helminth-induced CF in our study, suggesting that gut microbiota might be used as a therapeutic target for CF.

The potential for probiotics to be used in the management of CF is becoming an important research topic. In our study, we found that the *Akkermansia muciniphila* in *Verrucomicrobia* were more abundant in the intestine from Ts-infected mice using 16S rRNA amplicon sequencing and metagenomic sequencing. Similar result was observed in a systematic analysis of changes in intestinal flora caused by Ts infection [23]. One of the possible reasons for this observation could be that enteric helminth infection can increase epithelial mucus production [42], allowing *Akkermansia* residing in the mucus layer to flourish. Regrettably, no data is available for the potential association between induction of *Akkermansia* and enteric helminth infections. Interestingly, *A*. *muciniphila*, as a member of the gut microbiome, has been proposed as a next-generation probiotic. Oral gavage with *A*. *muciniphila* ameliorates the relative expression of fibrosis in the livers [43]. Our results showed that daily oral gavage with this probiotic is sufficient to reduce the severity of helminth-induce CF. CF is often secondary to underlying myocardial diseases. Specific circulating metabolites could contribute to the risk of pathological cardiovascular responses owing to alterations in the microbiota and their metabolites [38]. *A*. *muciniphila* has been also shown to improve metabolic outcomes, including vascular function [44,45]. Inflammatory signaling is closely involved in the progression of cardiac fibrosis. The pro-inflammatory cytokines are significantly elevated in many myocardial pathological conditions associated with fibrosis [46]. Treatment with *A muciniphila* substantially reduced the expression of pro-inflammatory cytokines including TNFα, IL-6 and IL-1β [44,45,47], indicating that the effect of *A muciniphila* is attributed to its anti-inflammatory activity.

Pasteurized *A*. *muciniphila* is more efficient than the alive bacterium and has proven safety and efficacy in numerous studies in mice and in a proof-of-concept study in humans [48]. In our study, we demonstrated that this pasteurized probiotic intervention attenuated helminth-induced CF via TLR2. It was also observed that the TLR2-interacting protein of pasteurized *A*. *muciniphila* reduces energy metabolism and improves insulin resistance [26]. In addition, TLR2 has a critical role in the acceleration of CF via infections caused by a major periodontal bacterium (*Porphyromonas gingivalis*) [49]. These data are different from our results, which showed that deficiency of TLR2 did not affect helminth-induced CF. This finding is not surprising considering that TLR2 recognize bacteria-associated molecular patterns [50], while TLR2 deficiency did not interfere the Ts burden in mice [51]. Another study shows that *A*. *muciniphila* could attenuate an inflammatory response associated with activation of the AMP-activated protein kinase (AMPK) and inhibition of the Nuclear Factor-Kappa B (NF-κB) signaling pathway via TLR2 [52]. And *A*. *muciniphila* triggers the production of anti-inflammatory IL-10 in a TLR2-dependent manner [53]. These studies indicate that the interaction between *A*. *muciniphila* and TLR2 involved in anti-inflammatory immune activation may be indispensable for protection against helminth-induced CF.

Notably, it is reported that diffuse and focal myocardial fibrosis occurs in the heart of patients with COVID-19. The autopsy result of COVID-19 patients revealed focal myocardial fibrosis in some cases [54,55]. Although respiratory failure is the primary cause of death, CF may also contribute to overall morbidity and mortality of COVID-19 patients [56]. Emerging preclinical and clinical studies indicate that pathogenesis and disease outcomes of severe acute respiratory syndrome coronavirus 2 (SARS-CoV-2) infection may be associated with altered gut microbiota [57]. Interestingly, the relative abundance of *Akkermansia* are higher in COVID-19 patients and K18-hACE2 mice infected by SARS-CoV-2 [58,59]. Others have shown that the abundance of *Akkermansia* positively correlated with infection of another virus influenza H7N9 and the beneficial effect of *A*. *muciniphila* on the pathogenesis and severity of H7N9 infection has been proved [60]. Future research exploring the effect of this probiotic in SARS-CoV-2 infection and related CF would be valuable.

Taken together, our findings suggest that the gut microbiota is involved in the development of helminth-induced CF. Treatment of pasteurized *A*. *muciniphila* could positively influence the outcomes of helminth-induced CF via TLR2. Several issues remain unresolved. The beneficial biomolecules of this probiotics are unknown. Further studies are needed to approve the positive effects of its derivatives on CF. While tremendous strides are currently being made in our understanding of *Akkermansia* function, much work remains to be done in other models of CF in mice or humans. In addition, it is also recommended to confirm the results observed in the experiments on germ-free mice.

## Materials and methods

### Ethical statement

All animal studies and the breeding process were carried out in accordance with guidelines approved by the Animal Welfare and Research Ethics Committee of Jilin University. C57BL/6J wild-type (WT) mice (female, 4–6 weeks old) were purchased from the Liaoning Changsheng Biotecnology co., Ltd., China. C57BL/6J TLR2 knockout (KO) mice (female, 6–8 weeks old) were purchased from the Nanjing University Model Animal Research Centre (Nanjing, China). All animals were maintained on standard rodent chow with water supplied ad libitum and with a 12/12 h light/dark cycle during the experimental period. The protocol was approved by the Institutional Animal Care and Use Committee of Jilin University.

### Helminth

The *T*. *spiralis* isolate (ISS534) was obtained from a naturally infected domestic pig in Henan Province in China. Briefly, *T*. *spiralis* muscle larvae (ML) were recovered from Wistar rats orally infected with 4000 infective larvae at 35 days post infection (dpi). To establish the model of helminth-induced cardiac fibrosis (CF), six-week-old male C57BL/6J mice were gavaged with 250 *T*. *spiralis* ML.

### Helminth infection with antibiotics treatment

To evaluate the role of gut microbiota in helminth-induced CF, mice were randomized into 3 groups (n = 6): (1) the control (Con) group were untreated, (2) the *T*. *spiralis* (Ts) group were infected with 250 ML, (3) the group infected with 250 Ts ML were administrated orally (dissolved in autoclaved water) with a cocktail of antibiotics (ampicillin 0.25 g/L, neomycin 0.25 g/L, metronidazole 0.25 g/L and vancomycin 0.125 g/L) daily from -14 to 14 dpi to deplete the gut microbiota. All the mice were euthanized using CO_2_ at 14 dpi.

### Fecal microbiota transplantation of helminth-altered gut microbiota

To explore whether helminth-altered gut microbiota cause CF, fecal microbiota transplantation (FMT) from helminth-infected mice to the control mice were performed. Mice were randomized into 2 groups (n = 6): (1) the control (Con) group were untreated, (2) the group received FMT from Ts-infected (14 dpi) mice (FMT-Ts) after administration orally (dissolved in autoclaved water) with a cocktail of antibiotics (ampicillin 0.25 g/L, neomycin 0.25 g/L, metronidazole 0.25 g/L and vancomycin 0.125 g/L) daily from -28 to 0 dpi. The process of FMT was performed as previously described [61]. Briefly, fresh fecal samples were collected from Ts-infected mice at 14 dpi and mixed with sterile PBS (1 fecal pellet/1 mL PBS) and homogenized immediately. The sample was centrifuged (100 x g, 5 minutes, 4°C), and the supernatant was used for transplantation. The supernatant (200 μL/mouse) was administered by gavage to recipient mice daily for 14 days. Control mice were treated with an equal volume of PBS. All the mice were euthanized using CO_2_.

### Fecal microbiota transplantation of healthy gut microbiota

To investigate whether the intervention on gut microbiota may be beneficial for helminth-induced CF, FMT from healthy mice to Ts-infected mice were performed. Mice were randomized into 2 groups (n = 6): (1) the Ts group were gavaged with 250 *T*. *spiralis* ML, (2) the Ts-infected group received FMT from healthy mice (FMT-H) after administration orally (dissolved in autoclaved water) with a cocktail of antibiotics (ampicillin 0.25 g/L, neomycin 0.25 g/L, metronidazole 0.25 g/L and vancomycin 0.125 g/L,) daily from -28 to 0 dpi. Briefly, fresh fecal samples were collected from healthy mice and mixed with sterile PBS (1 fecal pellet/1 mL PBS) and homogenized immediately. The sample was centrifuged (100 x g, 5 minutes, 4°C), and the supernatant was used for transplantation. The supernatant (200 μL/mouse) was administered by gavage to recipient mice infected with Ts daily for 14 days. Ts-infected control mice were treated with an equal volume of PBS. All the mice were euthanized using CO_2_.

### *Akkermansia muciniphila* administration

To confirm the effect of the dominant probiotic on helminth-induced CF, *Akkermansia muciniphila* administration were performed as described previously [51]. Briefly, *A*. *muciniphila* (ATCC BAA-835) was cultured in brain heart infusion media (BD Bioscience) supplemented with 0.4% mucin from porcine stomach (Sigma) and maintained in an anaerobic incubator using the GasPak 100 system (BD Bioscience) at 37°C. Cultures were centrifuged, the culture pellet was suspended in anaerobic PBS. Pasteurized *A*. *muciniphila* (PAm) was prepared by pasteurization for 30 min at 70°C. Alive *A*. *muciniphila* (AAm) or PAm was orally administered to mice (8 × 10^8^ CFU per dose) daily from 15 to 28 dpi. In addition, PAm was administered to wild-type (WT) or TLR2 KO mice orally (8 × 10^8^ CFU per dose) daily from 15 to 28 dpi. Mice were sacrificed using CO_2_ asphyxiation at 28 dpi.

Mice were weighed and recorded after euthanizing using CO_2_. Fecal specimens were snap-frozen in liquid nitrogen and stored at -80°C until further testing. At the time of euthanasia, the heart was immediately removed and weighed. Apical tissue was removed and fixed in 4% paraformaldehyde solution, and serum was also collected and stored at -80°C until further testing. Muscle larvae were recovered and counted at 28 dpi.

### Echocardiographic

Mice were subjected to transthoracic echocardiography using a high-frequency six ultrasound system equipped with a variable frequency 8–16 MHz probe. The animals were anesthetized with isoflurane (5%) in an inhalation chamber and maintained anesthetized with a dose of 1.25% isoflurane. The parasternal short-axis view was obtained at the level of the papillary muscles. Left ventricle (LV) internal dimensions at diastole/systole (LVIDd/LVIDs) and LV anterior/posterior wall thickness (LVAW/LVPW) were measured and used to calculate the ejection fraction (EF). Echocardiographic acquisition and echocardiographic data analysis were performed by an observer blinded to treatment.

### Heart gene expression

The mRNA expression levels of collagen 1 (Col1), α-SMA and TGF-β in heart were measured using real-time polymerase chain reaction as described previously [62]. The primers for Col1 were as follows: 5′-TCCAAAGGAGAGCGGTAA-3′ and *5′-GACCAGGACACA-3′*. The primers for α-SMA were as follows: 5′- TGCTGACAGAGGCACCACTGAA -3′ and 5′- CAGTTGTACGTCCAGAGGCATAG -3′. The primers for TGF-β were as follows: 5′- ACCTGCAAGACCATCGACAT -3′ and 5′- GGTTTTCTCATAGATGGCGT -3′. The relative mRNA expression levels of the target genes were normalized to those of the indicated housekeeping gene (GAPDH) (5′-TGAAGGGGTCGTTGATGG-3′ and 5′-AAATGGTGAAGGTCGGTGTG-3) and were quantified using the comparative Ct method and the formula 2^-ΔΔCT^.

### Masson staining

Paraffin sections were cut into 4-μm sections and stained with Masson’s trichrome to analyze the collagen present in heart samples. The tissue sections were baked at 60°C for 90 min; immersed in xylene (3 min × 3 times), absolute ethanol (3 min × 2 times), and 95% and 75% ethanol (each for 3 min); and flushed with running water. Then, the sections were stained with hematoxylin for 10 min, flushed with running water, differentiated with hydrochloric acid for several seconds, flushed with running water again and colorized to blue with ammonia for several minutes. Next, Ponceau-acid fuchsin, 12-molybdophosphoric acid solution and green staining solution were successively used to stain the sections for 4–20 s, 2–4 min, and 2–5 min, respectively. After washing with water and baking in an oven, the sections were sealed with neutral gum and observed and photographed under a microscope. In each section, five fields of view were chosen for observation under the microscope (×200).

### Immunohistochemistry

Immunohistochemistry (IHC) was performed as previously described [62]. Briefly, after antigen retrieval and blocking, slides were incubated overnight with their respective primary antibodies. The diluted (1:1000) primary antibody rabbit anti-mouse Col1a1 (Boster was added to the sections, and the slides were placed in a wet box and incubated at 4°C overnight. Rabbit serum was used as a negative control at the same time. After washing with PBS (2 min × 5 times), a biotin-labeled goat anti-rabbit secondary antibody (Beyotime Biotechnology) was added to the sections and incubated for 10 min, followed by two PBS washes. Then, a solution of horseradish peroxidase-conjugated streptavidin (Pierce, Rockford, IL) was added to the sections for 10 min of incubation, followed by washing with PBS (2 min × 5 times). Next, 3,3′-diaminobenzidine (DAB, ADI-950-211-0200, Assay Designs) was applied for color development for 1–2 min. Hematoxylin staining was employed to counterstain the nucleus for 1–1.5 min, and the slides were then washed slowly with running water. Afterward, the sections were differentiated by 0.1% hydrochloric acid and alcohol, colorized to blue, dehydrated and cleared. The sections were allowed to rest in ethanol (75%, 95%, 100%, separately), xylene I and xylene II for 1, 1, 1, 2, and 2 min, respectively. After being dried with xylene, the sections were sealed with neutral gum and photographed under a microscope.

### 16S ribosomal RNA gene sequencing

Total genome DNA from stool samples was extracted using QIAamp DNA Stool Mini Kit (Qiagen, Hilden, Germany). 16S ribosomal RNA (rRNA) gene amplification was performed using the primers (319F: 5′-ACTCCTACGGGAGGCAGCAG-3′; 806R: 5′-GGACTACHVGGGTWTCTAAT-3′) directionally targeting the V3 and V4 hypervariable region of the 16S rRNA gene. To differentiate each sample and yield accurate phylogenetic and taxonomic information, the gene products were attached with forward and reverse errorcorrecting barcodes. The amplicons were quantified after purification. Then, the normalized equimolar concentrations of each amplicon were pooled and sequenced on the MiSeq PE300 sequencing instrument (Illumina) using 2 × 300 bp chemistry according to the manufacturer’s specifications.

### Metagenomic sequencing

Faecal DNA was extracted using the QIAamp DNA Stool Mini Kit (Qiagen, Hilden, Germany). DNA integrity, sizes and concentrations were determined by agarose gel electrophoresis and NanoDrop spectrophotometry (NanoDrop, Germany). Sequencing libraries were constructed as previously described [63]. After library quality control, high-throughput sequencing was performed using the NovaSeq6000 platform (Illumina).

### Sequencing data analysis

As for 16S rRNA gene sequencing data (PRJNA917545), the raw data of 16S rRNA gene sequencing were analyzed using QIIME2 platform (v2020.2). In briefly, DADA2 plugin was used to filter the sequencing reads and to construct ASVs feature table. Taxonomic annotation of amplicon sequence variants (ASVs) representative sequences was performed with QIIME2. Alpha and beta diversity analyses were conducted using diversity plugin [64]. The α diversity and β diversity were calculated and displayed by QIIME and R software, respectively. Furthermore, we employed linear discriminant analysis (LDA) effect size (LEfSe) [62].

As for metagenomic sequencing data (PRJNA917023), raw sequencing reads were processed to obtain valid reads as previously described [63]. First, sequencing adapters were removed from sequencing reads using cutadapt (v1.9). Secondly, low-quality reads were trimmed by fqtrim (v0.94) using a sliding-window algorithm. Sample QC (quality control) was assessed using FastQC and Quast. Thirdly, reads were aligned to the host genome using bowtie2 (v2.2.0) to remove host contamination. Once quality-filtered reads were obtained, they were de novo assembled to construct the metagenome for each sample by IDBA-UD (v1.1.1). The clean reads were aligned to the database (V.202003, (ftp://ftp.ccb.jhu.edu/pub/data/kraken2_dbs/)) using Kraken2 software (V.2.1.1) and Braken software (V.2.5) to obtain species-level information. Based on the taxonomic profiling from the results of Kraken2, profiles at the species-level were selected for further analysis. The lowest common ancestor taxonomy of unigenes were obtained by aligning them against the NCBI NR database by DIAMOND (v0.9.14). Similarly, the functional annotation (KEGG [https://www.kegg.jp/]) of unigenes were obtained.

### Statistical analysis

The GraphPad Prism (version 8.0) package was used for statistical analysis and graphing. The Wilcoxon–rank sum test or Student’s t test for continuous variables were used to determine significant difference between two groups. Analysis of variance (ANOVA with Tukey adjustment for multiple comparisons) was used to compare the data among 3 or more groups. Statistical details for each experiment are stated in the figure legends with number of samples (or animals) “n” shown within the figures. Differences were considered significant at p < 0.05.

## Supporting information

S1 Fig The analysis of helminth burden after treatment of antibiotics (ABX), related to Fig 1. Muscle larvae of *T*.*spiralis* (Ts) were recovered from mice in each group and the burden of Ts were calculated. Data are shown as individual data points and mean ± SD. Data were compared by one-way ANOVA followed by Tukey multiple comparison tests. ns, not significant.
(DOCX)

S2 Fig Helminth-induced gut microbiota dysbiosis cannot result in cardiac fibrosis, related to Fig 3. (A) Experimental scheme of mice received fecal microbiota transplant (FMT) from Ts-infected mice. (B) Whole heart images of the controls (Con) (n = 6) and mice received FMT)of Ts-altered microbiota (FMT-Ts) (n = 6). Representative images are shown. (C) Heart mass-to-body weight ratio (HM/BW). (D and E) Ejection fraction (EF) and left ventricular weight (LVM) obtained by cardiac ultrasound. (F and G) qPCR analysis of Collagen-1 (Col1) and α-SMA expression in the heart tissue of mice. (H and I) Masson staining and Col1 immunohistochemistry results of heart tissues. Magnification, 200×. Scale bars, 100 μm. Representative images are shown. Data are shown as individual data points and mean ± SD. Statistical significance is calculated using paired student t-test. ns, not significant; *, p < 0.05; **, p < 0.01, ***, p <0.001, ****, p <0.0001.
(DOCX)

S3 Fig Enrichment analysis of differentially expressed genes in the KEGG pathway by metagenomics, related to Fig 4. The horizontal axis indicates the degree of enrichment (Rich factor), and the vertical axis indicates the enriched KEGG pathway; the size of the dots indicates the number of differentially expressed genes enriched in a KEGG pathway; the color of the dots indicates different p values; the Rich factor indicates the number of differentially expressed genes belonging to a KEGG pathway/the total number of genes belonging to this KEGG pathway. The larger the Rich factor, the higher the enrichment of the KEGG pathway.
(DOCX)

S4 Fig The analysis of helminth burden after treatment of *Akkermansia muciniphila*, related to Fig 5. Muscle larvae of *T*.*spiralis* (Ts) were recovered from mice in each group and the burden of Ts were calculated. Data are shown as individual data points and mean ± SD. Data were compared by one-way ANOVA followed by Tukey multiple comparison tests. ns, not significant.
(DOCX)

S1 Data Excel spreadsheet containing, in separate sheets, the data points presented in Figs 1–6 and S1–S4. (XLSX)
