# Supplementary material for: Akkermansia muciniphila participates in the host protection against helminth-induced cardiac fibrosis via TLR2
Source: PLoS Pathog. 2023 Oct 3;19(10):e1011683. doi: 10.1371/journal.ppat.1011683 (PMC10547169; doi:10.1371/journal.ppat.1011683)
Supplement: S2 Fig — (A) Experimental scheme of mice received fecal microbiota transplant (FMT) from Ts-infected mice. (B) Whole heart images of the controls (Con) (n = 6) and mice received FMT)of Ts-altered microbiota (FMT-Ts) (n = 6). Representative images are shown. (C) Heart mass-to-body weight ratio (HM/BW). (D and E) Ejection fraction (EF) and left ventricular weight (LVM) obtained by cardiac ultrasound. (F and G) qPCR analysis of Collagen-1 (Col1) and α-SMA expression in the heart tissue of mice. (H and I) Masson staining and Col1 immunohistochemistry results of heart tissues. Magnification, 200×. Scale bars, 100 μm. Representative images are shown. Data are shown as individual data points and mean ± SD. Statistical significance is calculated using paired student t-test. ns, not significant; *, p < 0.05; **, p < 0.01, ***, p <0.001, ****, p <0.0001. (DOCX) [file ppat.1011683.s002.docx]

**S2 Fig. Helminth-induced gut microbiota dysbiosis cannot result in cardiac fibrosis, related to Fig 3.**

(A) Experimental scheme of mice received fecal microbiota transplant (FMT) from Ts-infected mice.

(B) Whole heart images of the controls (Con) (n=6) and mice received FMT)of Ts-altered microbiota (FMT-Ts) (n=6). Representative images are shown.

(C) Heart mass-to-body weight ratio (HM/BW).

(D and E) Ejection fraction (EF) and left ventricular weight (LVM) obtained by cardiac ultrasound.

(F and G) qPCR analysis of Collagen-1 (Col1) and α-SMA expression in the heart tissue of mice.

(H and I) Masson staining and Col1 immunohistochemistry results of heart tissues. Magnification, 200×. Scale bars, 100 μm. Representative images are shown.

Data are shown as individual data points and mean ± SD. Statistical significance is calculated using paired student t-test. ns, not significant; *, p < 0.05; **, p < 0.01, ***, p <0.001, ****, p <0.0001.

**
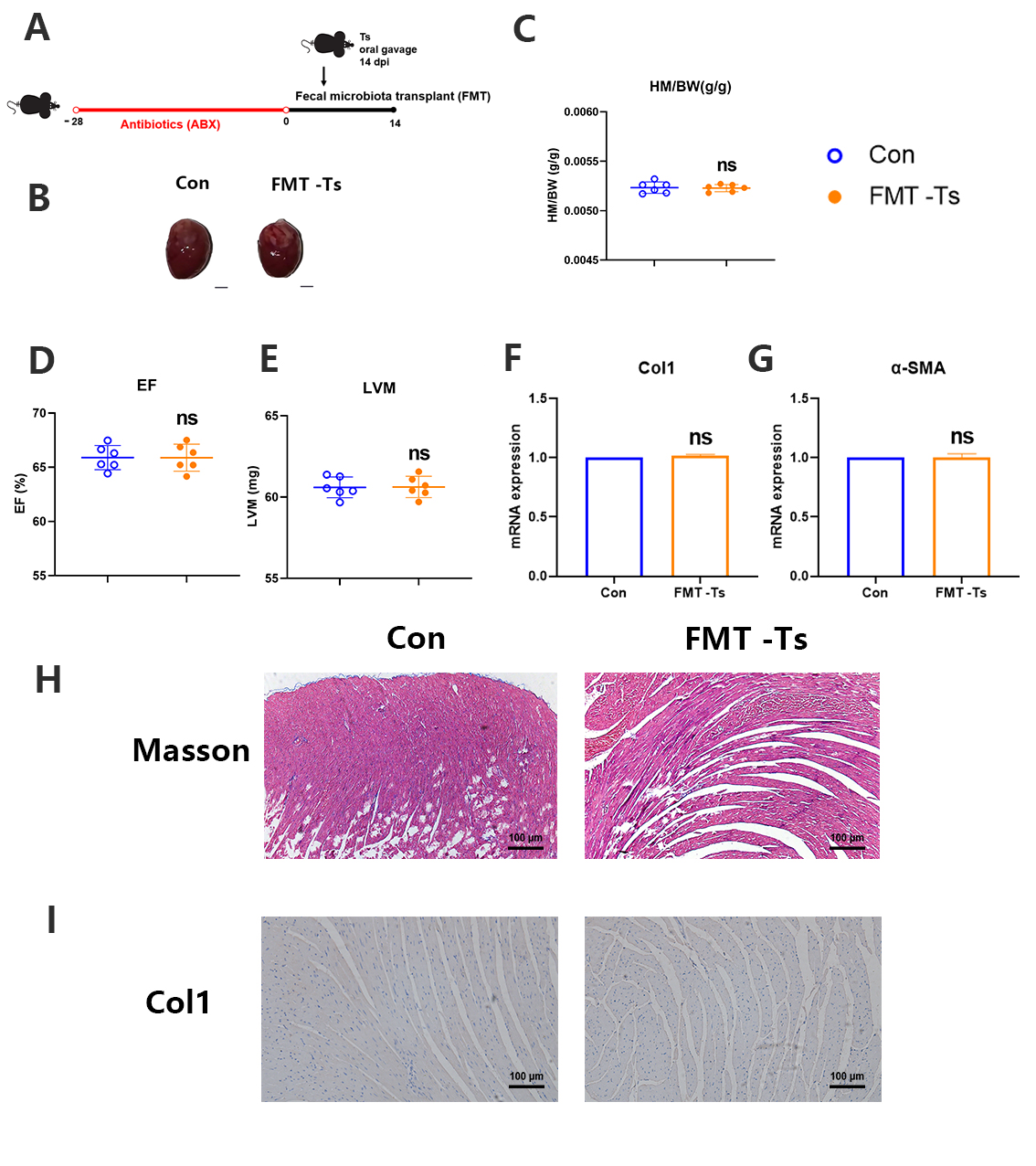
**
