# Supplementary material for: Akkermansia muciniphila participates in the host protection against helminth-induced cardiac fibrosis via TLR2
Source: PLoS Pathog. 2023 Oct 3;19(10):e1011683. doi: 10.1371/journal.ppat.1011683 (PMC10547169; doi:10.1371/journal.ppat.1011683)
Supplement: S3 Fig — The horizontal axis indicates the degree of enrichment (Rich factor), and the vertical axis indicates the enriched KEGG pathway; the size of the dots indicates the number of differentially expressed genes enriched in a KEGG pathway; the color of the dots indicates different p values; the Rich factor indicates the number of differentially expressed genes belonging to a KEGG pathway/the total number of genes belonging to this KEGG pathway. The larger the Rich factor, the higher the enrichment of the KEGG pathway. (DOCX) [file ppat.1011683.s003.docx]

**
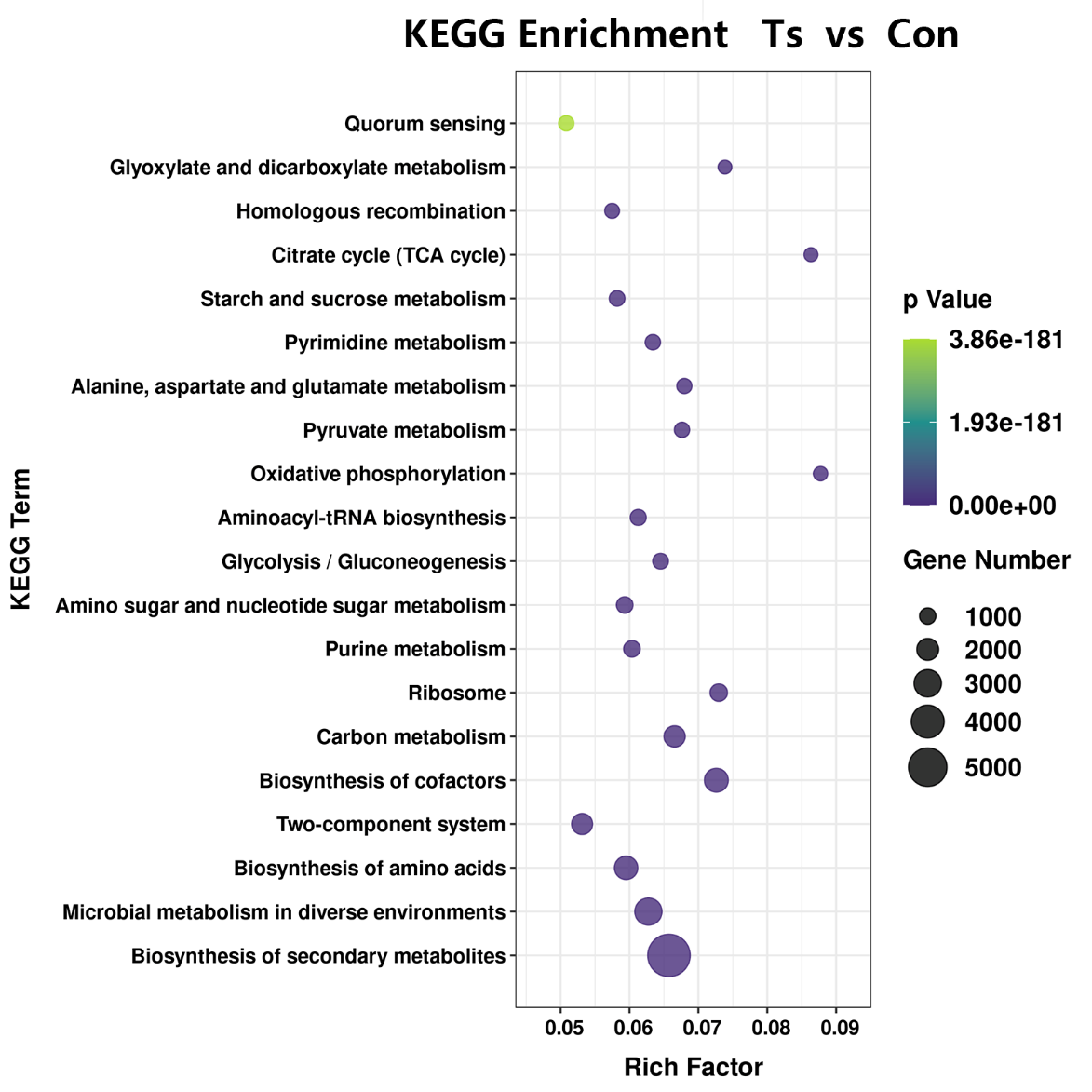
**

**S3 Fig. Enrichment analysis of differentially expressed genes in the KEGG pathway by metagenomics, related to Fig 4.**

The horizontal axis indicates the degree of enrichment (Rich factor), and the vertical axis indicates the enriched KEGG pathway; the size of the dots indicates the number of differentially expressed genes enriched in a KEGG pathway; the color of the dots indicates different p values; the Rich factor indicates the number of differentially expressed genes belonging to a KEGG pathway/the total number of genes belonging to this KEGG pathway. The larger the Rich factor, the higher the enrichment of the KEGG pathway.
