# Supplementary material for: Akkermansia muciniphila participates in the host protection against helminth-induced cardiac fibrosis via TLR2
Source: PLoS Pathog. 2023 Oct 3;19(10):e1011683. doi: 10.1371/journal.ppat.1011683 (PMC10547169; doi:10.1371/journal.ppat.1011683)
Supplement: S4 Fig — Muscle larvae of T.spiralis (Ts) were recovered from mice in each group and the burden of Ts were calculated. Data are shown as individual data points and mean ± SD. Data were compared by one-way ANOVA followed by Tukey multiple comparison tests. ns, not significant. (DOCX) [file ppat.1011683.s004.docx]

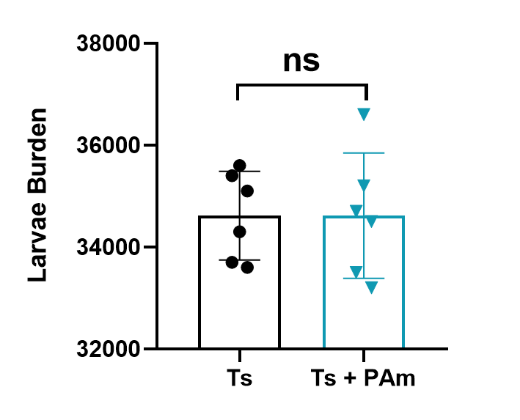


**S4 Fig. The analysis of helminth burden after treatment of *Akkermansia muciniphila*, related to Fig 5*.***

Muscle larvae of *T.spiralis* (Ts) were recovered from mice in each group and the burden of Ts were calculated. Data are shown as individual data points and mean ± SD. Data were compared by one-way ANOVA followed by Tukey multiple comparison tests. ns, not significant.
